# Introducing MCC-PS: a novel prognostic score for Merkel cell carcinoma

**DOI:** 10.3389/fonc.2024.1427740

**Published:** 2024-07-22

**Authors:** Nessr Abu Rached, Jürgen C. Becker, Anke S. Lonsdorf, Aric Keller, Ioannis A. Zeglis, Thilo Gambichler

**Affiliations:** ^1^ Skin Cancer Center, Department of Dermatology, Venereology and Allergology, Ruhr-University Bochum, Bochum, Germany; ^2^ International Centre for Hidradenitis Suppurativa/Acne Inversa (ICH), Department of Dermatology, Venereology and Allergology, Ruhr-University, Bochum, Germany; ^3^ Translational Skin Cancer Research, DKTK Partner Site Essen/Düsseldorf, West German Cancer Center, Dermatology, University Duisburg-Essen, Essen, Germany; ^4^ German Cancer Research Center (DKFZ), Heidelberg, Germany; ^5^ Medical Faculty Heidelberg, Department of Dermatology and National Center for Tumor Diseases (NCT), NCT Heidelberg, a Partnership between DKFZ and University Hospital Heidelberg, Heidelberg University, Heidelberg, Germany; ^6^ Department of Dermatology, University Hospital, Ruprecht-Karls University of Heidelberg, Heidelberg, Germany; ^7^ Department of Dermatology and Phlebology, Christian Hospital Unna, Unna, Germany; ^8^ Department of Dermatology, Dortmund Hospital gGmbH and Faculty of Health, Witten/Herdecke University, Dortmund, Germany

**Keywords:** Merkel cell carcinoma, prognostic score, MCC-PS, Merkel cell carcinoma prognosis score, death, relapse, biomarkers

## Abstract

**Introduction:**

Merkel cell carcinoma (MCC) is an aggressive skin cancer with a poor prognosis, which only improved with the introduction of immunotherapies. An MCC prediction model with high diagnostic accuracy is lacking. The aim was to develop an MCC prognostic score (MCC-PS) based on combinations of previously proposed risk factors.

**Methods:**

A multicentric, retrospective study was conducted to develop MCC-PS, which included age, neuron-specific enolase (NSE), C-reactive protein (CRP), creatinine, bilirubin, and international normalized ratio (INR). Creatinine, bilirubin, and INR were used to calculate the model of end-stage liver disease (MELD) score. A total of 98 patients were included in the study, including 36.7% with stage I according to American Joint Committee on Cancer 2018 (*n* = 36), 30.6% with stage II (*n* = 30), 25.5% with stage III (*n* = 25), and 7.1% with stage IV (*n* = 7). Survival data of MCC patients were correlated with selected laboratory parameters and risk factors. Primary endpoint was MCC-specific survival (MSS) and the secondary endpoint was progression-free survival. Several statistical methods were used to develop the prognostic score, including correlation analysis, Kaplan–Meier curves, Cox regression, and time-dependent receiver operating characteristic analysis.

**Results:**

The MCC-PS is based on the sum of the following baseline variables: elevated CRP (≥5.5 mg/l), elevated NSE (≥22.8 µg/l), MELD score ≥ 11, and age ≥ 75 years. An MELD score ≥ 11 was scored as 4 points, elevated NSE level as 3 points, elevated CRP level as 2 points, and age ≥ 75 years as 1 point. A high-risk group according to the MCC-PS was characterized by a score of 4 or more points. The high-risk group was associated with a worse prognosis than the low-risk group (1-year MSS 62%, 2-year 43.1%, 5-year 17.6% as compared to 1-year MSS 79.5%, 3-year 75%, 5-year 72%). Notably, the developed MCC-PS predicts MCC outcome measures with high accuracy (3-year MSS: area under the curve (AUC) 0.934, sensitivity 87.5% and specificity 82.2%; 5-year MSS: AUC 0.93, sensitivity 89% and specificity 82%).

**Conclusion:**

MCC-PS is the first prognostic score predicting MCC outcome with a high accuracy based on five easily available laboratory parameters and patient’s age. An MCC-PS of 4 or more indicates a high-risk patient associated with a poor prognosis.

## Introduction

Merkel cell carcinoma (MCC) is an aggressive skin cancer with a poor prognosis. The development of immunotherapies improved the prognosis of MCC (1). In recent years, more studies conducted on the use of neoadjuvant and adjuvant immunotherapy in cutaneous melanoma that delivered impressive results (2, 3). The relevance of adjuvant immunotherapy for MCC has recently reported (4). Adjuvant immunotherapy with nivolumab led to an absolute risk reduction of 9% (1-year disease-free survival, DFS) and 10% (2-year DFS) (4). Currently, there is no score that accurately predicts the prognosis of MCC, making it difficult to decide which patients should receive adjuvant therapy. Thus, a prognostic score distinguishing between high- and low-risk patients would improve the management of MCC.

Clonal integration of Merkel cell polyomavirus (MCPyV) can be found in MCC in about 80% of cases (5, 6). Known risk factors for the development of MCC include chronic UV exposure, immunosuppression, and advanced age (7, 8). Serological biomarkers have recently been reported to be associated with prognosis of MCC patients. For example, pan-immune-inflammation value, neuron-specific enolase (NSE), and model of end-stage liver disease (MELD) score have a prognostic potential in MCC patients (9–11). However, these biomarkers individually have a relatively low specificity and sensitivity, making them less suitable for clinical practice. In the present study, we developed an MCC prognostic score (MCC-PS) that well represents disease recurrence and MCC-specific mortality by combining patient’s age and five blood parameters [bilirubin, creatinine, international normalized ratio (INR), C-reactive protein (CRP), NSE; all values at initial diagnosis].

## Patient and methods

### Design and setting

A multicentric, retrospective diagnostic accuracy study was conducted in Germany (Bochum, Heidelberg, and Unna) to develop an MCC prognosis score. The study was conducted in accordance with ethical principles of the Declaration of Helsinki (12). This retrospective non-interventional study was approved by institutional ethics review board at the Ruhr University Bochum (ethics vote: #16–5985).

### Patients and data collection

All patients with complete laboratory data and available survival data were included in this retrospective study. There were otherwise no exclusion criteria. Laboratory parameters assessed included a complete blood count, CRP, aspartate transferase (AST), alanine transaminase (ALT), NSE, lactate dehydrogenase (LDH), bilirubin, creatinine, and INR. The MELD score, which is associated with MCC outcome, was calculated using INR, bilirubin and creatinine as follows (10).


. MELD=3.78 x ln(serum bilirubin {mg/dL}) + 11.2×ln(INR) + 9.57×ln(serum creatinine {mg/dL}) + 6.43

In addition, further demographic and MCC-specific data was collected. Clinical data included age, sex, immunosuppression, comorbidities, as well as Charlson Comorbidity Index (13). MCC-specific data include stage according to American Joint Committee on Cancer (AJCC) 2018, presence of lymph node metastases, presence of distant metastases, tumor thickness, MCPyV status, location of primary tumor, presence of high-risk region, and MCC outcome [progression-free survival (PFS), MCC relapse, MCC-specific survival]. All patients were treated according to the German guideline for MCC (1).

### Sample size

Sample size calculation was in accordance with the methods of Riley et al., 2019 and 2020 (14, 15). MCC-specific death was the primary endpoint of the prognostic model. We used an estimated event rate of 0.4 and an *r*-squared value of 0.15. A sample size of at least 50 patients was required for four potential predictors. This procedure can be used to calculate the sample size online at https://mvansmeden.shinyapps.io/BeyondEPV/.

### Data analysis

Data are presented as absolute figures, percentages, medians, and interquartile ranges (IQRs) as indicated. Non-parametric analysis, including Spearman rank correlation and Mann–Whitney *U* test, was used to determine simple associations between the different variables and MCC outcome measures. Univariable and multivariable statistical methods, including Spearman rank correlation, Mann–Whitney *U* tests, Cox regression, log-rank test, and Kaplan–Meier curves, were used to determine the associations between the different variables and the outcome of MCC. To determine the cutoff value, a ROC analysis with determination of the area under the curve (AUC) and the Youden index was carried out. We also performed a time-dependent ROC analysis to assess the diagnostic accuracy of the MCC-PS at multiple time points as it is more effective than the standard ROC analysis (16, 17). The model and weighting were based on the regression coefficient, hazard ratio (HR), *z*-score, and *P*-value. Using the results of the Cox proportional hazards model, a Youden index was calculated for each combination of prognostic factors (CRP, NSE, age, and MELD score). The best Youden index was used for the MCC-PSS. Statistical analysis was carried out using IBM SPSS (IBM Corporation, version 29.0, New York, 2022) and R software (R Core Team, version 4.3.1, Vienna, Austria, 2022). Statistically significant level was set at *p*< 0.05.

## Results

### Patient’s and disease-specific characteristics

Overall, we collected retrospective data on long-term survival of MCC with a complete laboratory workup at the time of diagnosis from 98 patients. The median age was 77 years (IQR, 71–83), and more than 56% of MCC patients were aged 75 years or older (**Table 1**). The sex distribution was almost equal with 47 female (48%) and 51 male patients (52%). According to the 2018 AJCC, most MCC cases were stages I and II, accounting for 67.3% of total cases (*n* = 66, respectively). Stage III accounted for 25.5% of cases (*n* = 25), while stage IV was the least common, accounting for only 7.1% of cases (*n* = 7). Median PFS was 11.5 months, with an IQR of 5.8–32.5 months. The median MCC-specific survival (MSS), representing the time from diagnosis to MCC-specific death, that is, from histological diagnosis to death due to MCC progression or complications, was 19.5 months, with an IQR spanning from 10 to 49 months. Furthermore, the study reveals that 31.6% of patients experienced MCC-specific mortality, emphasizing the seriousness of MCC.

**Table 1:** Patient’s characteristics and disease-specific characteristics of 98 patients with MCC.^a^.

Parameters	Values
Sex, *n* (%)	
female	47 (48)
male	51 (52)
Age, median (IQR), y	77 (71–83)
Age ≥ 75 years	56 (57.1)
Tumor stage at diagnosis (according AJCC 2018)	
Stage I, *n* (%)	36 (36.7)
Stage II, *n* (%)	30 (30.6)
Stage III, *n* (%)	25 (25.5)
Stage IV, *n* (%)	7 (7.1)
Laboratory data at initial diagnosis	
NSE level ≥ 22.8 µg/l, *n* (%)	39 (39.8)
CRP ≥ 5.5 mg/l, *n* (%)	32 (32.7)
MELD-score ≥ 11	21 (21.4)
Outcome data	
Progression-free survival, median (IQR), months	11.5 (5.8–32.5)
MCC relapse, *n* (%)	43 (43.9)
MCC-specific survival, median (IQR), months	19.5 (10–49)
MCC specific death, *n* (%)	31 (31.6)

n, absolute number of patients; IQR, interquartile range; CRP, c-reactive protein; NSE, neuron-specific enolase; MELD, model of end stage liver disease; y, years; AJCC, American Joint Committee on Cancer; MCC, Merkel cell carcinoma.

a Total number of MCC patients: 98.

### Relationship between MCC outcome measures and prognostic variables

To determine the optimal cutoff values for the different variables, we performed ROC analyses and determined the Youden index. Univariate analyses showed that MCC-specific death was associated with increased CRP, increased NSE, increased MELD score ≥ 11, and age ≥ 75 years (*p*< 0.001; 0.002;< 0.001; 0.047, respectively). In addition, the variable MCC-specific death in months was associated with increased CRP, increased MELD score, increased NSE, and age ≥75 years (*p*< 0.1). MCC relapse was also associated with the variables elevated CRP, elevated NSE, elevated MELD score (*p*< 0.05). There was no correlation between the outcome data and the other laboratory values AST, ALT, LDH, bilirubin, creatinine, and INR (*p* > 0.05). **Supplementary Figure S1** shows the ROC curves of the individual variables CRP, NSE, MELD score, and age in relation to recurrences and MCC-specific deaths. Between time points, the AUC values of the ROC curves vary (**Supplementary Figure S1**). For example, the AUC value for elevated CRP and MCC-specific death varies from 0.647 to 0.719 depending on the time point (**Supplementary Figure S1A**).

### Score development of the MCC-PS

To improve diagnostic accuracy, we developed a score based on four variables, that is, CRP, NSE, MELD score, age. Optimal cutoff values were determined by ROC analysis and the Youden index. CRP ≥5.5, NSE level≥22.8, MELD score ≥11, and age ≥75 years were found to be the optimal cutoff values (Youden index = 0.406; 0.257; 0.343; 0.192, respectively). Next, we used the Cox proportional-hazards regression model to assess the effect of the four variables in predicting MCC-specific death (**Table 2**). The overall model showed statistical significance (χ^2^ = 38.17; *p*< 0.001), indicating its ability to predict outcomes. Elevated CRP showed a regression coefficient of 1.28 (95% CI 0.49–2.06), corresponding to an HR of 3.58 (95% CI 1.64–7.83; *p* = 0.001). Elevated NSE levels had a regression coefficient of 1.28 (95% CI 0.5–2.06), resulting in a high HR of 3.6 (95% CI 1.66–7.83; *p* = 0.001). An MELD score ≥11 was associated with a regression coefficient of 1.38 (95% CI 0.62–2.13) and an HR of 3.97 (95% CI 1.87–8.45; *p*< 0.001). Finally, individuals aged ≥ 75 years had a regression coefficient of 1.0 (95% CI 0.18–1.83) and a HR of 3.73 (95% CI: 1.2–6.21; *p* = 0.017). *Z*-scores and *p*-values indicate the statistical significance of each variable’s contribution to the model. Based on the results (**Table 2**), MELD score (≥11) was weighted fourfold, NSE (≥22.8) threefold, CRP (≥5.5) twofold, and age (≥75 years) onefold.

**Table 2:** Cox proportional hazard model to detect the effect of the four variables; the overall model was significant (Chi-Quadrat = 38.17; *p*< 0.001).

	Regression coefficient (95% CI)	Hazard ratio, HR, (95% CI)	*Z*- score	*P*-value
CRP level ≥ 5.5 mg/l	1.28 (0.49–2.06)	3.58 (1.64–7.83)	3.2	0.001
NSE level ≥ 22.8 µg/l	1.28 (0.5–2.06)	3.6 (1.66–7.83)	2.4	0.001
MELD-Score ≥11	1.38 (0.62–2.13)	3.97 (1.87–8.45)	3.58	< 0.001
Age ≥ 75 years	1.0 (0.18–1.83)	3.73 (1.2–6.21)	2.7	0.017

CRP, c-reactive protein; NSE, neuron-specific enolase; MELD, model of end stage liver disease; CI, confidence interval.

### Calculation of the Merkel cell carcinoma score

**Figure 1:**
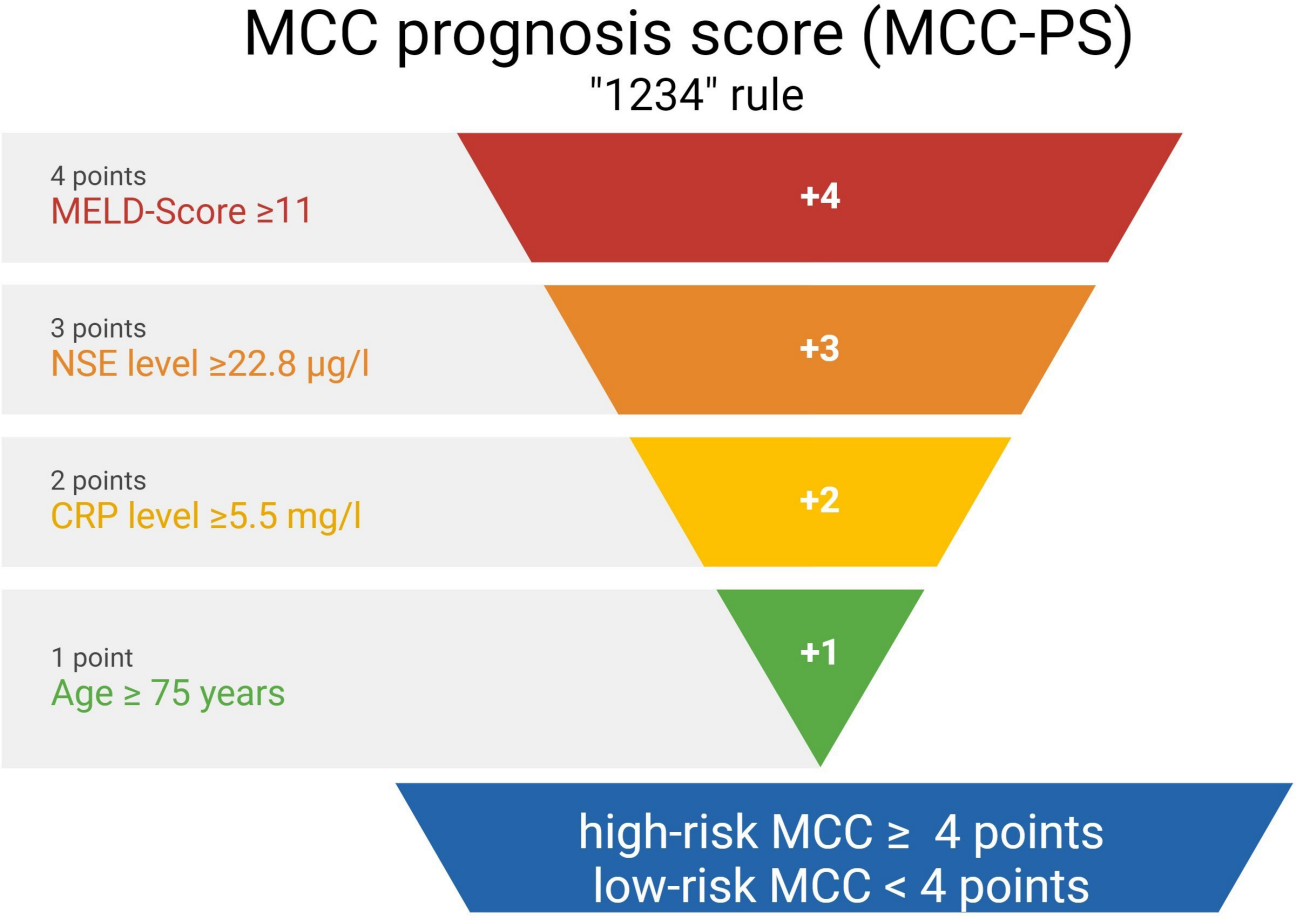
The figure shows the calculation of the Merkel cell carcinoma score (MCC-PS) based on five laboratory values and the patient age at initial diagnosis.

**Figure 2:**
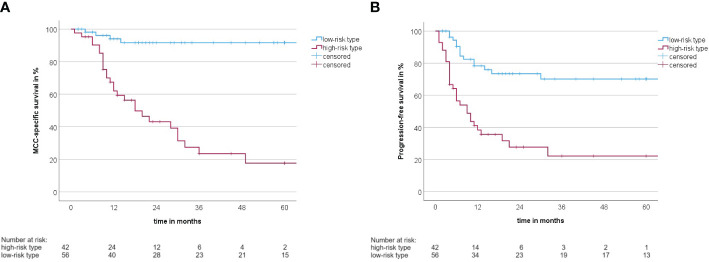
**(A)** Kaplan–Meier curve with MCC-specific survival rate between the high-risk type and the low-risk type according to the MCC-PS (log-rank test: *p*< 0.001). **(B)** Kaplan–Meier curve with progression-free survival between the high-risk type and the low-risk type according to the MCC-PS (log-rank test: *p*< 0.001).

A summary of how the MCC-PS is calculated is shown in **Figure 1** and **Supplementary Table S1**. Five common laboratory parameters (bilirubin, INR, creatinine, CRP, and NSE) and age at first diagnosis are required to determine MCC-PS. INR, bilirubin, and creatinine are used to calculate the MELD score. MELD ≥11 scores 4 points for the MCC-PS, NSE ≥ 22.8 3 points, CRP ≥ 5.5 2 points, and age ≥75 years 1 point. These four components (CRP, NSE, MELD-score, and age) sum to the MCC-PS. A total MCC-PS score ≥4 indicates the high-risk group and a total score of less than 4 indicates the low-risk group. A significantly worse prognosis is associated with high risk according to MCC-PS (**Figure 2**). For patients classified to be at high risk, the 1-year MSS was 62%, but it drops to 23.5% at the 3-year mark. Conversely, the low-risk group shows significantly better survival, with a 1-year MSS of 94.5% and maintaining a robust 92.1% MSS throughout the 5-year period (**Supplementary Table S2**). In terms of PFS, a similar pattern emerges. High-risk patients have a 1-year PFS of 38.3%, but this decreases to 22.2% beyond the 3-year mark. In contrast, low-risk patients display notably improved PFS, with a 1-year rate of 79.5% and a consistent 72% PFS over the 5-year duration.

### Prognostic accuracy of the Merkel cell carcinoma score

**Figure 3:**
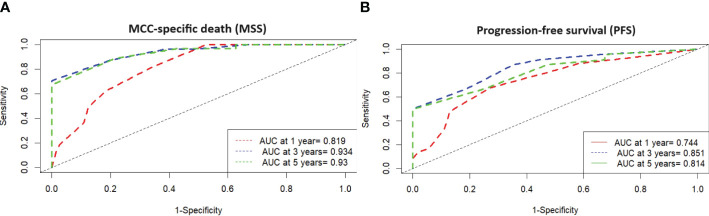
**(A)** Time-dependent ROC analysis with MSS and high-risk type according MCC-PS shows that the development of the prognostic score has improved the diagnostic accuracy compared to the individual variables. **(B)** Time-dependent ROC analysis with PFS and high-risk type according MCC-PS shows that the development of the prognostic score has improved the diagnostic accuracy compared to the individual variables.

**Figure 4:**
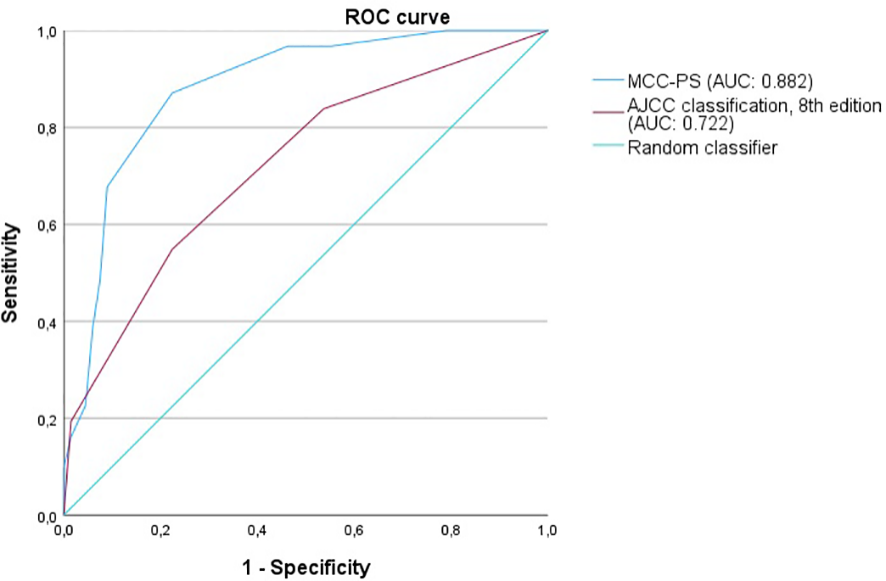
The figure shows the ROC curves for predicting MCC-specific death (MSS). MCC-PS predicts MSS better than the AJCC 8th edition classification.

ROC analysis of all data (MCC-specific death and MCC-PS) showed a sensitivity of 87.1% and specificity of 77.6% with a cutoff value of 3.5 and a Youden index of 0.647. To determine diagnostic accuracy, we performed time-dependent ROC analyses. The results of the time-dependent ROC analysis are presented in **Table 3** and **Figure 3**. When comparing the prognostic accuracy of MCC-PS with the AJCC 2018 classification, MCC-PS performed better (**Figure 4**): for MCC-specific death, the sensitivity was 54.8%, specificity 77.6%, and the Youden index 0.325 (cutoff value of 2.5).

**Table 3:** Time-dependent ROC analysis of diagnostic accuracy of MCC-PS for MCC-specific death and MCC relapse.

Diagnostic accuracy for	Time	AUC	Sensitivity	Specificity	PPV	NPV
MCC-specific death	*T* = 12	0.819	0.812	0.64	0.33	0.938
	*T* = 24	0.841	0.845	0.7	0.547	0.913
	*T* = 36	0.934	0.875	0.822	0.753	0.914
	*T* = 48	0.939	0.882	0.84	0.783	0.916
	*T* = 60	0.93	0.89	0.8	0.763	0.909
MCC relapse	*T* = 12	0.744	0.678	0.709	0.592	0.779
	*T* = 24	0.786	0.677	0.794	0.739	0.74
	*T* = 36	0.851	0.664	0.863	0.832	0.716
	*T* = 48	0.854	0.664	0.895	0.865	0.723
	*T* = 60	0.814	0.664	0.8	0.771	0.701

t, time in months; AUC, area under the curve; PPV, positive predictive value; NPV, negative predictive value.

## Discussion

Calculated prognostic scores and models play an important role in medicine. For example, the Child-Pugh Score is a composite score that represents the prognosis in liver cirrhosis (18). Here we report that the MCC-PS shows high specificity (64%–84%) and sensitivity (81.2%–89%) for MCC-specific death. Indeed, compared with single, MCC unspecific variables, the combination of four variables into an overall score increased the accuracy for predicting MCC outcome, for example, the specificity for predicting recurrences was also high (70.9%–89.5%), albeit at a lower the sensitivity (66.4%–67.8%). Thus, the MCC-PS is particular suitable for clinical use with respect to MCC-specific death.

As the MCC-PS is calculated from five common laboratory parameters (bilirubin, INR, creatinine, NSE, CRP) and the patient’s age, the score is easy to apply and does not require further invasive and/or costly tests. Bilirubin, INR, and creatinine are used to calculate the MELD, which was shown to be an independent prognostic risk factor, but with low sensitivity (10). The latter was improved by adding more variables. Interestingly, the MCC-PS was better than the AJCC stages (for MCC-specific death: AUC of 0.824, sensitivity of 87.1%, specificity of 77.6%, Youden index of 0.647 vs. AUC of 0.618, sensitivity of 100%, specificity of 37.9%, Youden Index of 0.379) in predicting MCC-specific death and disease recurrence. Interestingly, additionally including tumor thickness, tumor diameter, stage IV, and the presence of lymph node metastases did not improve the accuracy of the MCC-PS.

The development of the MCC-PS is important for clinical practice and future therapy, since adjuvant immunotherapy will become increasingly important in MCC (19). MCC-PS allows differentiation of high- and low-risk patients. It is important to stratify by risk group in future clinical trials. Survival data for immunotherapy varies widely in some trials. For example, Avelumab demonstrated an objective response rate of 33%–40% in clinical trials, a 24-month PFS of 26%, and a 24-month OS of 36% (20, 21). Part B of the JAVELIN Merkel 200 trial showed that avelumab monotherapy resulted in a significant long-term OS (4-year OS rate of 38%) in patients with MCC (22). PD-1 inhibitor pembrolizumab showed higher objective response rates (56%), 24-month PFS (48%), and 24-month OS (69%) (23). It is possible that response rates and MCC outcome measures are significantly higher by using the stratification according to MCC-PS. In addition, it will be interesting to see whether patients of the high-risk group are more likely to have adverse events. Since some laboratory values are elevated in the high-risk group, this may indicate poorer liver, coagulation, and kidney function when compared to low-risk patients. MCC-PS offers new perspectives in the treatment and diagnosis of MCC. However, validation of the MCC-PS in a prospective study is warranted.

In the present study, we used a multicentric approach to reduce bias. However, our analysis is limited by its retrospective design and a relatively small sample size. Nevertheless, the low incidence and our calculation of the case size implies that the number of cases is sufficient.

## Conclusion

MCC-PS is the first prognostic score that robustly predicts MCC outcome measures with a high accuracy based on patient’s age and five common laboratory parameters. An MCC-PS value of 4 or more identity’s a high-risk group, which is associated with a poorer prognosis. Determination of MCC-PS may be useful for evaluating new drug trials. Validation of the MCC-PS in a prospective study is warranted.

## Data availability statement

The original contributions presented in the study are included in the article/**Supplementary Material**. Further inquiries can be directed to the corresponding author.

## Ethics statement

The studies involving humans were approved by institutional ethics review board at the Ruhr-University Bochum. The studies were conducted in accordance with the local legislation and institutional requirements. The participants provided their written informed consent to participate in this study.

## Author contributions

NA: Conceptualization, Formal Analysis, Funding acquisition, Investigation, Methodology, Project administration, Resources, Software, Supervision, Visualization, Writing – original draft, Writing – review & editing. JB: Formal Analysis, Investigation, Methodology, Writing – original draft, Writing – review & editing. AL: Investigation, Resources, Writing – original draft, Writing – review & editing. AK: Investigation, Resources, Writing – original draft, Writing – review & editing. IZ: Investigation, Resources, Writing – original draft, Writing – review & editing. TG: Formal Analysis, Investigation, Methodology, Supervision, Writing – original draft, Writing – review & editing.

## Supplementary material

Further tables and figures can be found in the Supplementary Material.
