# Supplementary material for: Introducing MCC-PS: a novel prognostic score for Merkel cell carcinoma
Source: Front Oncol. 2024 Jul 22;14:1427740. doi: 10.3389/fonc.2024.1427740 (PMC11298473; doi:10.3389/fonc.2024.1427740)

Supplementary Material

**Supplementary Table 1.** This Table shows MCC-specific survival (MSS) and progression-free survival (PFS) in patients with MCC. The prognosis worsens with a higher score on the Merkel cell carcinoma prognosis score (MCC-PS). According to the MCC-PS, a high-risk type of MCC is present with at least 4 points.

|  | 0  point | 1  point | 2  points | 4  points | 5  points | 10  points |
| --- | --- | --- | --- | --- | --- | --- |
|  | low-risk type | | | high-risk type | | |
| 1-year  MSS | 100 % | 76.5 % | 70.7 % | 62.0 % | 53.5 % | 33.3 % |
| 2-years  MSS | 100 % | 64.8 % | 58.8 % | 43.1% | 34.6 % | 0 % |
| 3-years  MSS | 100 % | 52.5 % | 44.5 % | 23.5 % | 5.8 % | 0 % |
| 4-years  MSS | 100 % | 52.5 % | 44.5 % | 17.6 % | 5.8 % | 0 % |
| 5-years  MSS | 100 % | 49.2 % | 40.4 % | 17.6 % | 5.8 % | 0 % |
|  | | | | | | |
|  | **0**  **point** | **1**  **point** | **2**  **points** | **4**  **points** | **5**  **points** | **10**  **points** |
|  | low-risk type | | | high-risk type | | |
| 1-year  PFS | 85.1 % | 57 % | 49.4 % | 38.3 % | 30.9 % | 0% |
| 2-years  PFS | 85.1 % | 48.4 % | 39.1 % | 27.7 % | 18.5 % | 0% |
| 3-years  PFS | 85.1 % | 42.9 % | 32.3 % | 22.2 % | 9.3 % | 0% |
| 4-years  PFS | 85.1 % | 42.9 % | 32.3 % | 22.2 % | 9.3 % | 0% |
| 5-years  PFS | 85.1 % | 42.9 % | 32.3 % | 22.2 % | 9.3 % | 0% |

**Supplementary Table 2.** This Table shows MCC-specific survival (MSS) and progression-free survival (PFS) in patients with MCC compared to high-risk and low-risk MCC.

|  | Low-risk type  MCC-PS < 4 | High-risk type  MCC-PS ≥ 4 |  | Low-risk type  MCC-PS < 4 | High-risk type  MCC-PS ≥ 4 |
| --- | --- | --- | --- | --- | --- |
| 1-year  MSS | 94.5% | 62 % | 1-year  PFS | 79.5 % | 38.3 % |
| 2-years  MSS | 92.1 % | 43.1 % | 2-years  PFS | 75 % | 27.7 % |
| 3-years  MSS | 92.1 % | 23.5 % | 3-years  PFS | 72 % | 22.2 % |
| 4-years  MSS | 92.1 % | 23.5 % | 4-years  PFS | 72 % | 22.2 % |
| 5-years  MSS | 92.1 % | 17.6 % | 5-years  PFS | 72 % | 22.2 % |

**Supplementary Figure 1.**

Figure 1a shows the time dependent ROC curves with the state variable MCC specific death and with the different test variables at time points t=12, 36, 60 months; a) test variable: CRP ≥5.5, b) test variable: NSE ≥ 22.8, c) test variable: MELD score ≥ 11, d) test variable: age≥75.


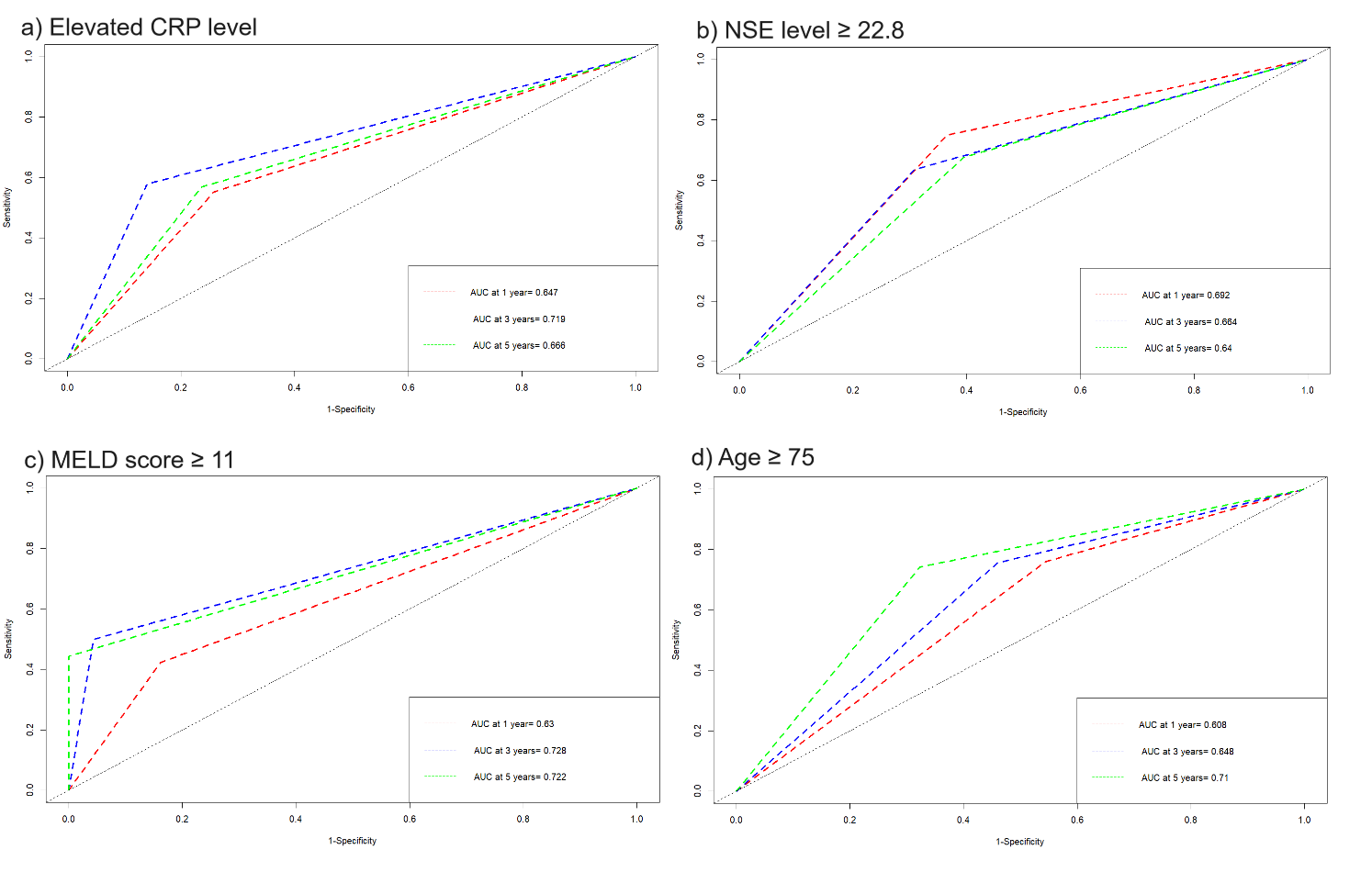


Figure 1b shows the time dependent ROC curves with the state variable MCC relapse and with the different test variables at time points t=12, 36, 60 months; a) test variable: CRP ≥5.5, b) test variable: NSE level ≥ 22.8, c) test variable: MELD score ≥ 11, d) test variable: age≥75.


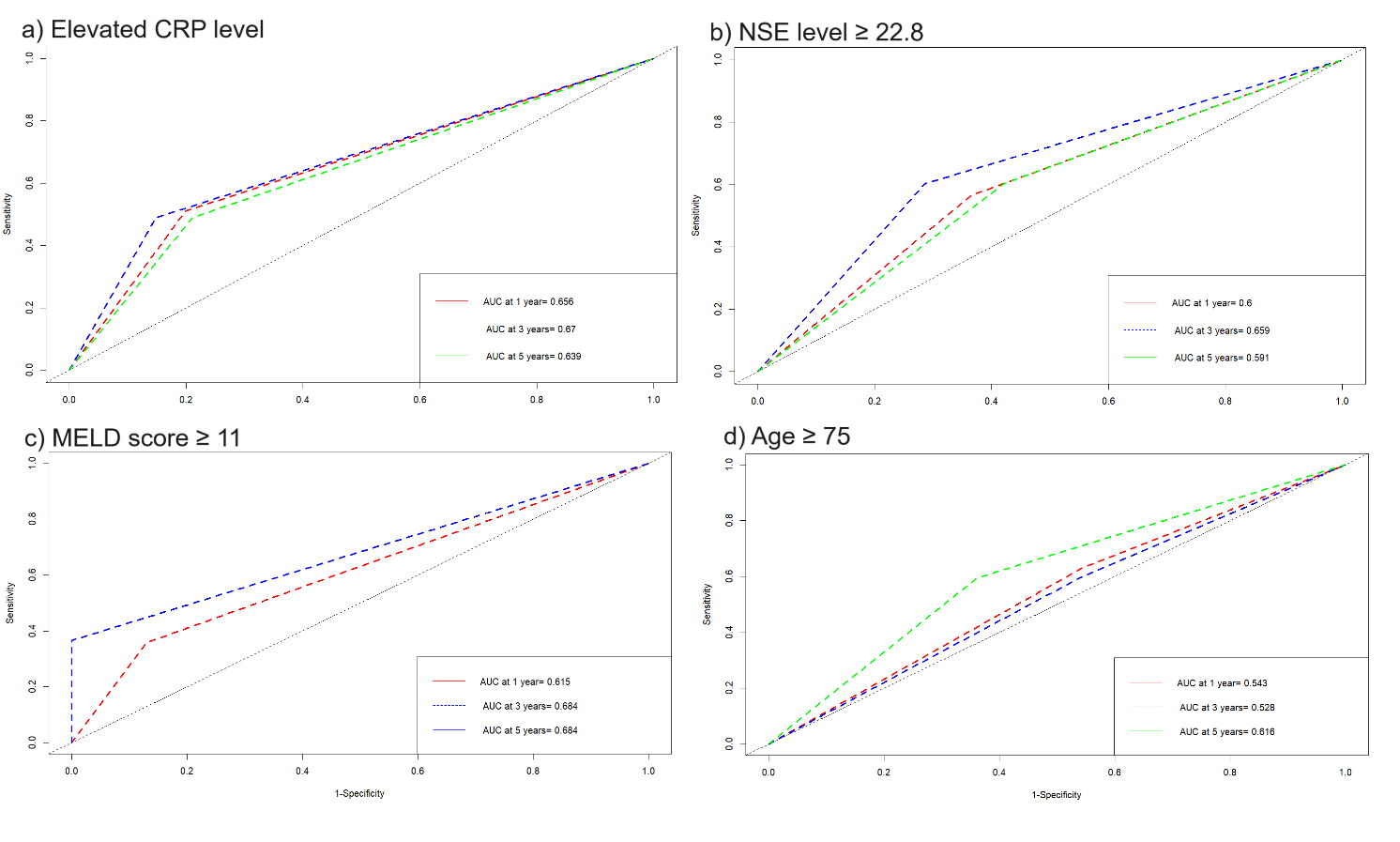

Supplement: Supplementary file 1 [file DataSheet_1.docx]
